# Evaluation of radiation dose for inferior vena cava filter placement during pregnancy: A comparison of dosimetry and dose calculation software

**DOI:** 10.1002/acm2.13884

**Published:** 2022-12-22

**Authors:** Yuta Matsunaga, Tomonobu Haba, Masanao Kobayashi, Shoichi Suzuki, Yasuki Asada, Koichi Chida

**Affiliations:** ^1^ Department of Imaging Nagoya Kyoritsu Hospital Nagoya Aichi Japan; ^2^ Department of Radiological Technology Faculty of Health Sciences Tohoku University Graduate School of Medicine Sendai Miyagi Japan; ^3^ School of Health Sciences Fujita Health University Toyoake Aichi Japan

**Keywords:** fetal dose, pregnancy, entrance surface dose

## Abstract

Numerous medical conditions are associated with pregnancy in women, including pulmonary thromboembolism, which can be fatal. An effective treatment of this condition is the positioning of an inferior vena cava filter (IVC‐F) under the guidance of X‐ray imaging. However, this procedure involves the risk of high radiation exposure to pregnant women and fetuses. Moreover, there are no published reports comparing the values of fetal dose, received during IVC‐F placement in pregnant women, determined using dose calculation software and actual measurements. To address this issue, we compared the fetal radiation dose and entrance surface dose (ESD) for pregnant women for gestation periods of 6 and 9 months based on software calculations and actual measurements. The ESD and fetal doses were estimated for a pregnant woman for gestation periods of 6 and 9 months during IVC‐F placement. For actual measurements, one pregnant model phantom was constructed using an anthropomorphic phantom, and two custom‐made different‐sized abdomen phantoms were used to simulate pregnancy. The custom‐made abdomen phantoms were constructed using polyurethane. For software calculations, the software utilized a set of anatomically realistic pregnant patient phantoms. The ESD estimated using the software was consistent with the measured ESD, but the fetal dose estimations were more complicated due to fetal positioning. During fetal dose evaluation using software calculations, the user must carefully consider how much of the fetal length is in the irradiation field to prevent underestimation or overestimation. Despite the errors, the software can assist the user in identifying the magnitude of the dose approaching critical limits.

## 1 INTRODUCTION

Pregnant women may develop various conditions during pregnancy such as infections, high blood pressure, and abnormal glucose metabolism. Pulmonary thromboembolism (PTE) is a serious condition with a high case fatality rate and possibility of recurrence. Thrombosis is more likely to occur during pregnancy than in a normal condition because blood coagulates more easily during pregnancy, and the iliac vein and inferior vena cava are compressed by the enlarged uterus. According to the Japan Association of Obstetricians and Gynecologists report,^1^ 24 (7%) of the 338 maternal deaths from January 2010 to June 2018 were PTE cases. On an average, two or more pregnant women die from PTE annually in Japan. Approximately 90% of PTE embolic sources are formed from thrombi in the lower extremities or pelvic veins. In addition to anticoagulant therapy, an inferior vena cava filter (IVC‐F) should be positioned under the guidance of X‐ray imaging to prevent fatal PTE. Many patients benefit greatly from interventional radiology (IVR) procedures^2^, ^3^, ^4^, ^5^, ^6^ such as IVC‐F placement. However, a major disadvantage associated with these procedures is radiation exposure to patients. The biological effects of radiation are of two types: stochastic (such as radiation‐induced cancer) and deterministic (such as erythema). Currently, one of the most important problems in IVR for general adults is the occurrence of the deterministic effects of radiation because the number of case reports documenting patient skin injuries from IVR is increasing.^7^, ^8^, ^9^, ^10^, ^11^, ^12^ However, the location of the fetus is close to the position where the IVC‐F is generally placed. The radiation risk to the fetus in pregnant women undergoing IVC‐F is more critical. The fetal dose and organ dose have not been evaluated in pregnant women during IVC‐F placement.

Recently, radiation dose in the field of medicine has been lowered through advanced equipment and studies investigating diagnostic reference levels.^13^, ^14^, ^15^, ^16^, ^17^, ^18^ Accordingly, several dose reports^19^, ^20^, ^21^, ^22^, ^23^, ^24^, ^25^, ^26^ have been published for the examination of pregnant women or fetuses using X‐ray imaging. Matsunaga et al.^20^ evaluated the radiation dose received during CT examinations of pregnant women and fetuses and found a difference between the values obtained using commercial software and actual radiation dose measurement results. The use of commercially available dose calculation software tools, which are mostly based on Monte Carlo calculations, is widespread throughout the medical field.^14^, ^21^, ^27^, ^28^, ^29^, ^30^, ^31^ The majority of these Monte Carlo‐based software packages use stylized patient phantoms with overly simplified anatomies.^27^ Moreover, these software tools only consider average‐size patients and ignore other common groups, such as pregnant patients, which limits their clinical utility. In a previous study by Matsunaga et al.,^20^, ^21^ the radiation dose required for pregnant women and fetuses was evaluated using stylized patient phantoms with overly simplified anatomies. VirtualDose‐IR (Virtual Phantoms Inc., Albany, New York, USA) is a cloud‐based software tool for assessing and reporting patient organ doses, which was developed to facilitate the calculation of patient doses during IR procedures. The software is based on a comprehensive patient organ dose database that utilizes extensive Monte Carlo simulations using a set of anatomically realistic patient phantoms including pregnant women at different gestational stages. Huo et al.^32^ evaluated the dose differences between VirtualDose‐IR results and other software programs results for pregnant patients. However, there are no published reports that compare the fetal dose received during IR procedures in pregnant women when the values are determined using dose calculation software and actual measurements.

To address this deficiency, we compared the fetal radiation dose and entrance surface dose (ESD) for pregnant women of different gestational stages using these two approaches during IVC‐F placement.

## 2 MATERIALS AND METHODS

### 2.1 Actual measurements

In this study, ESD and fetal dose were estimated during IVC‐F placement using pregnant model phantoms of different sizes and real‐time dosimeters (RTDs).

**FIGURE 1:**
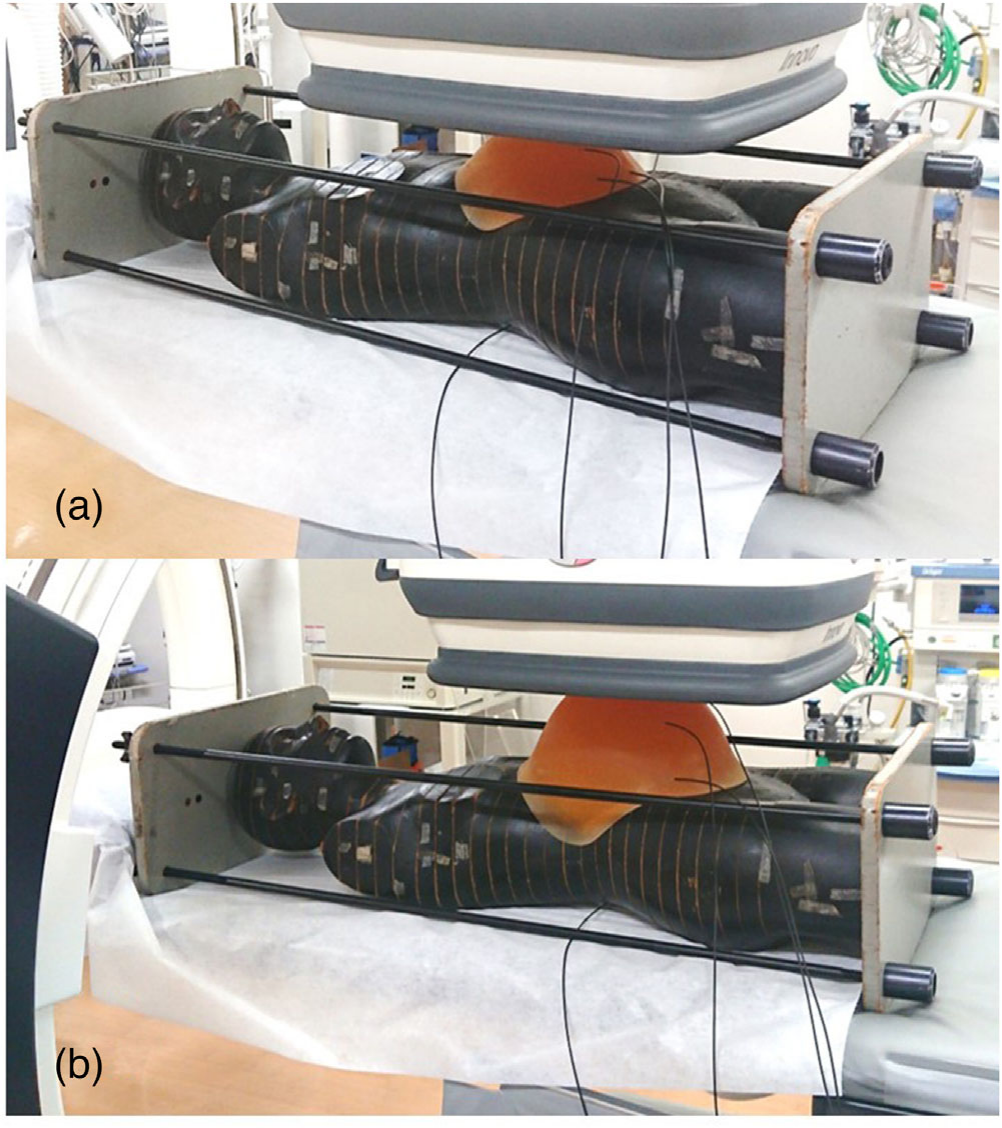
Phantom used to represent a pregnant woman during actual measurements. (a) Small pregnant phantom; (b) Large pregnant phantom.

One pregnant model phantom was constructed using an anthropomorphic phantom (Alderson Rando phantom), and two custom‐made abdomen phantoms of different sizes were used to simulate pregnancy (Kyoto Kagaku Co. Ltd., Kyoto, Japan) (Figure 1). The size of the two phantoms approximately corresponded to the size of a pregnant woman's abdomen in the second or third trimesters of gestation; hereafter, these are referred to as the small and large pregnant phantoms.^23^ The custom‐made abdomen phantoms were constructed using polyurethane. The abdominal circumference values of the umbilicus of the small and large pregnant phantoms were 80 and 95 cm, respectively. The large abdomen phantom was used along with the small abdomen phantom by placing it on top of the latter. Both abdomen phantoms were designed with holes to facilitate the implantation of dosimeters.

The angiography X‐ray unit used in this study was a monoplane interventional imaging system (GE Healthcare, Chicago, IL, USA) with a 20‐cm mode flat‐panel detector. In this study, the exposure parameters were determined based on the dose area product (DAP) data obtained during IVC‐F placement in adults from only one single hospital. The tube voltage, tube current, cine‐pulse width, and filtration were automatically adjusted by the X‐ray system. Table 1 presents the exposure parameters.

**TABLE 1:** Exposure parameters used for the actual measurements

	Tube voltage (kV)	Tube current (mA)	Pulse width (ms)	Filtration (mm Cu)	DAP (cGy cm^2^)	Projection
Small pregnant phantom	84 80^a^	317.2	64	0.1	1906.5	PA
Large pregnant phantom	97 90^a^	283	90	0.1	1850.5	PA

^a^ Values entered into the dose calculation software.

**FIGURE 2:**
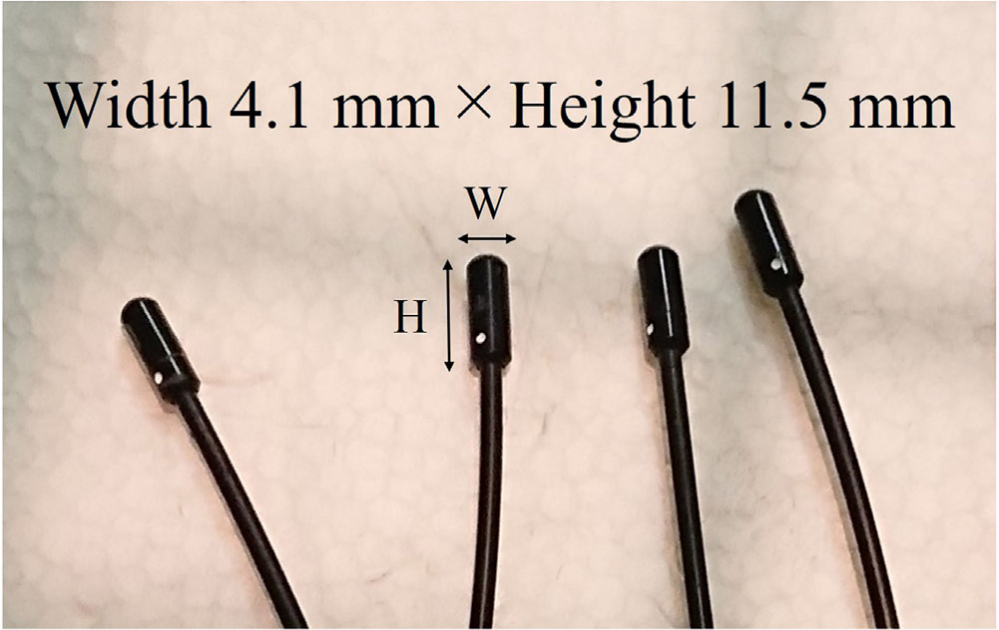
Sensor size.

**FIGURE 3:**
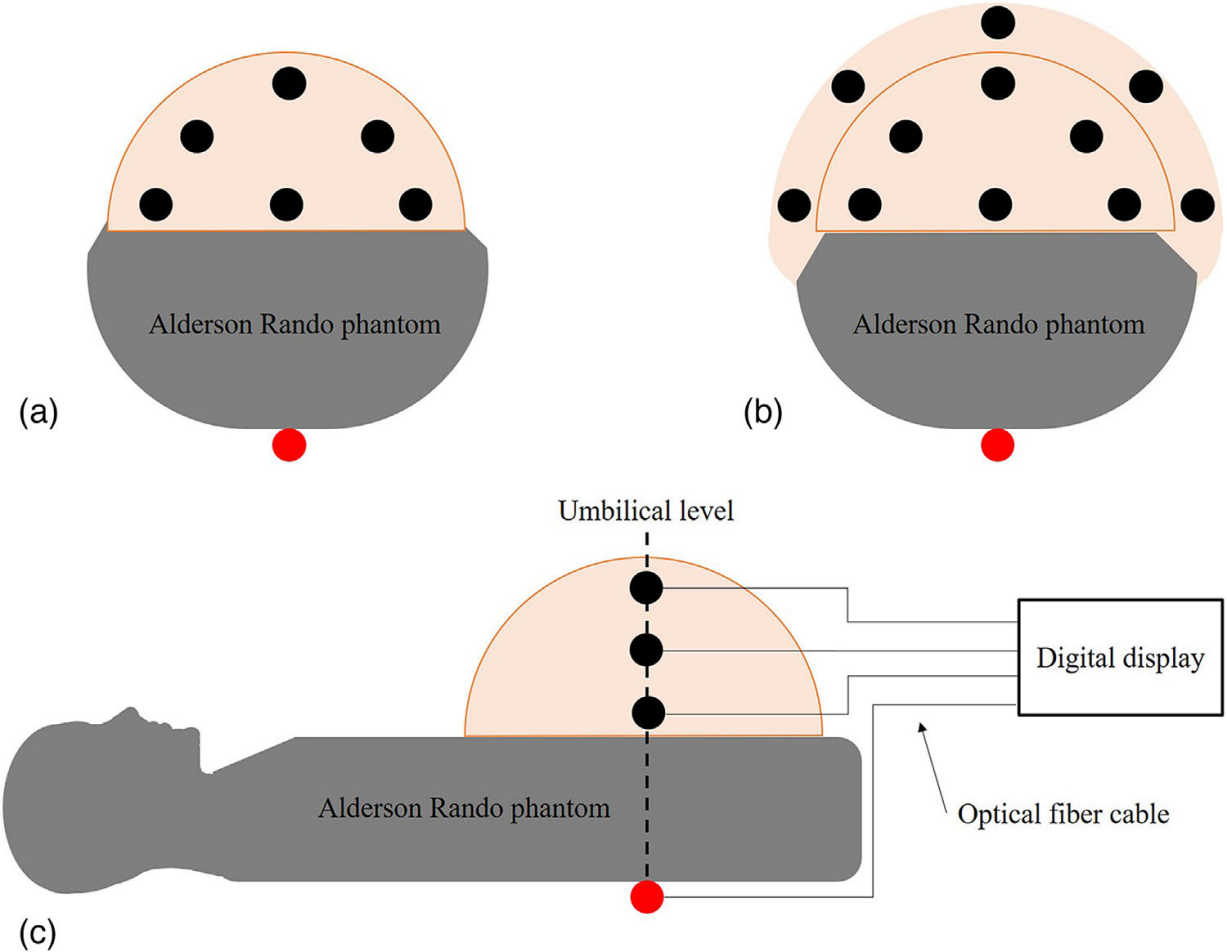
A graphic illustration depicting measurement placements at the umbilical level. (a) Small pregnant phantom (b) Large pregnant phantom (c) An illustration showing the side view of the phantom. Black dots show placements of RTDs for fetal doses. Red dots show placement of RTD for ESD.

The absorbed radiation doses during IVC‐F placement were measured using RTDs (RD‐1000, TORECK Corporation, Kanagawa, Japan).^12^, ^22^, ^23^, ^33^, ^34^, ^35^ They consisted of sensors (scintillator: Y_2_O_2_S: Eu, Sm) having a cylindrical shape with dimensions φ4.1 × 11.5 mm (Figure 2), an optical fiber cable, a photodiode, and a digital display that included the power supply. RTDs for fetal doses were implanted at 6 and 11 points at the umbilical level of the small and large pregnant phantoms, respectively (Figure 3). RTDs for ESD measurement were located at a single point at the entrance surface of the small and large pregnant phantoms. Each measurement was performed three times to reduce random error.

On RTD, the manufacturer (TORECK Corporation, Kanagawa, Japan) quoted that the guaranteed range of X‐ray detection accuracy was 0.1–2000 mGy/min, and measurement errors were 0.1–100 mGy/m: ±10% + 0.5 (mGy/min) and 100.1–2000 mGy/m: ±10%. There was no angular dependence in this study. Although there was no energy dependence from 34 to 40 keV, the maximum −20% energy dependence from 28 to 33 keV was reported in the previous study.^34^, ^36^

Dose calibration of the RTDs for each tube voltage diagnostic X‐ray beam was performed based on a comparison to an ionization chamber dosimeter (9015, Radcal Corporation, Monrovia, CA, USA) with a 6‐cm^3^ thimble ionization chamber (10 × 5‐6, Radcal Corporation) that was traceable to a national standard. The chamber dosimeters and RTDs were placed freely in air adjacent to each other in the irradiated field at equal distance from the X‐ray focus. The correction factor was determined using this calibration procedure. Each radiation dose in the 6 or 11 measured doses from RTDs was calculated by multiplying the value obtained from each RTD based on the correction factor by the ratio of the mass‐energy absorption coefficient for air and the coefficient for soft tissue. The fetal dose was an average measurement of all the radiation doses in the 6 or 11 measured doses from RTDs because it was assumed to irradiate the entire body of the fetus, as described in previous studies.^19^, ^22^ In addition, accurate ESD could be obtained by correcting the RTD values based on an ionization dosimeter using calibration factors. Each calibration was performed three times to reduce random error.

### 2.2 Estimation based on the dose calculation software

In this investigation, ESD and fetal doses were estimated for pregnant women at 6 and 9 months gestation using the software VirtualDose‐IR (dose calculation software). The physical pressure by the pregnant uterus constitutes one of the largest risks in the venous thromboembolism. The targets of the study were 6 and 9 months of gestation, developing a large pregnant uterus throughout the pregnancy. A set of whole‐body mesh‐based computational phantoms in the dose calculation software were used to represent patients in IVR clinical treatments. Those mesh‐based phantoms were converted into voxel‐based phantoms using an in‐house voxelization algorithm to adopt Monte Carlo dose calculations. Masses and heights of the voxelized 6 and 9 month pregnant women phantoms used in the dose calculation software were 66.6 kg, 163.5 cm, and 72.4 kg, 163.5 cm, respectively. Voxel size in those phantoms was 2 × 2 × 2 mm. To calculate peak skin doses (PSDs), some air‐filled voxel arrays were defined along the skin entrance to recorded air kerma over the irradiation area. The maximum air kerma was identified and converted to skin dose using available Hp (0.07)/Ka conversion factors. Moreover, a 2.15 mm Al equivalent thickness operating bed was considered. The above explanation is based on the previous study^32^ describing the development and testing of VirtualDose‐IR for calculating organ doses and effective doses of patients undergoing IVR procedures.

Because the selectable tube voltages in this dose calculation software were 70, 80, and 90 kV, the estimations in the pregnant woman at 6 and 9 months gestation were selected as 80 and 90 kV, respectively. All other exposure parameters using the dose calculation software were identical to those used for the small and large pregnant phantoms in the actual measurements (Table 1). The beam direction used in these calculations was posterior‐anterior. The dose calculation software used DAP as the machine output measurement for dose calculation. The PSD calculated using the dose calculation software was substituted with ESD, and the field of view in all the cases was 20 × 20 cm.

In a previous study,^23^ the anatomical geometry of the small and large pregnant phantoms in actual measurements were made based on the shape, fetal location, and CT number in actual pregnant patients. Because the shapes of the tube current modulation in the z‐axis for the pregnant model phantom satisfactorily simulated that of a patient,^23^ the 6 and 9 month phantoms are similar to the actual pregnant patient. The anatomical geometry of pregnant model phantoms can be used as the phantoms using the dose calculation software.

## 3 RESULTS

### 3.1 Comparison between the actual measurement and the dose calculation software value for the estimated radiation dose

Table 2 presents a comparison between the fetal dose and ESD for the actual measurements and dose calculation software values. At 6 months, the fetal dose and ESD values were 0.84 and 0.87 smaller, respectively, for the software‐based values compared with those acquired based on actual measurements. However, at 9 months, the fetal dose and ESD values were 0.52 and 0.83 smaller, respectively, for the dose calculation software compared with the actual measurements.

**TABLE 2:** Comparison of fetal dose and ESD estimated based on actual measurements and the dose calculation software

	6 month gestational stage	Small pregnant phantom	Difference		9 month gestational stage	Large pregnant phantom	Difference	
	Software (mGy)	Dosimetry (mean ± SD) (mGy)	Ratio	Software–dosimetry	Software (mGy)	Dosimetry (mean ± SD)(mGy)	Ratio	Software–dosimetry
Fetal dose	2.39	2.84 ± 1.47	0.84	−0.45	1.89	3.63 ± 1.95	0.52	−1.74
ESD	98.64	113.37 ± 0.23	0.87	−14.73	102.05	122.62 ± 0.95	0.83	−20.57

### 3.2 The organ‐specific doses for pregnant women and fetuses using the dose calculation software

Table 3 presents the results for organ‐specific doses for a pregnant woman and fetus obtained using the dose calculation software for gestations of 6 and 9 months. The organ doses for pregnant women (gonads, kidneys, and skin) within the irradiation field were higher than 1 mGy. The other organ doses for pregnant women outside of the irradiation field were less than 1 mGy. The organ doses of the fetus (brain, bone, soft tissue, and total) for 6 months gestation were 4.04 mGy, 9.02 mGy, 1.57 mGy, and 2.39 mGy, respectively, and the corresponding values for 9 months were 3.70 mGy, 8.07 mGy, 0.79 mGy, and 1.89 mGy, respectively.

**TABLE 3:** Organ‐specific doses for pregnant woman and fetus estimated using the dose calculation software for 6 and 9 months gestation

		6 month gestational stage	9 month gestational stage
		(mGy)	(mGy)
Pregnant woman			
	Adrenals	0.43	0.46
	Bone surface	9.11	8.78
	Brain	0.00	0.00
	Breast	0.02	0.02
	Extrathoracic region	0.01	0.01
	Gonads	14.87	14.73
	Heart	0.04	0.04
	Kidneys	2.52	2.38
	Liver	0.15	0.17
	Lung	0.08	0.09
	Esophagus	0.02	0.03
	Pancreas	0.97	0.98
	ESD	98.64	102.05
	Skin	1.15	1.04
	Spleen	0.2	0.23
	Stomach	0.21	0.24
	Thymus	0.01	0.02
Fetus			
	Fetal brain	4.04	3.70
	Fetal skeleton	9.02	8.07
	Fetal soft	1.57	0.79
	Fetus total	2.39	1.89
Cumulative air kerma		160.67	163.67

## 4 DISCUSSION

Both the fetal dose and ESD values obtained at 6 months gestation using the dose calculation software were similar compared with the values obtained via actual measurements. Likewise, the ESD obtained at 9 months using the dose calculation software was approximately equal to that obtained via actual measurements. However, the fetal dose at this gestation period was 0.52 smaller when the dose calculation software was used compared with the values obtained via actual measurements. This difference is associated with the difference in the assumed fetus length in actual measurements and when the dose calculation software is used. In the case of actual measurements, the assumed fetus length for 6 and 9 months gestation was mostly within the irradiation field. Although the assumed fetus length at 6 months was almost within the irradiation field for the dose calculation software, at 9 months, a part of the fetus was outside of the irradiation field because of growth. In a previous study,^32^ the organ dose errors for adults were within 30% when the values obtained for the VirtualDose‐IR software were compared with actual measurements. The largest difference was observed for organs that were subjected to relatively low doses that were outside the irradiation field. The dosage outside of the irradiation field is significantly lower than that within the field. Thus, the total fetal dose that was determined using the dose calculation software at 9 months was lower than that obtained via actual measurements. When fetal dose evaluation is performed using VirtualDose‐IR, the user should be careful to ensure that how much of the fetal length is in the irradiation field to prevent underestimation or overestimation.

In this study, it was determined that the ESD of pregnant patients was smaller compared to the individual cumulative air kerma (CAK) values. The CAK value is the air kerma measured at a point located 15 cm from the center of the isocenter of an IVR X‐ray unit on the central beam towards the focus (interventional reference point).^37^ This should facilitate an assessment of the dose delivered to the patient's skin and provide a better indication of deterministic effects. However, as the X‐ray tube irradiates the patient from different angles or positions, it is likely to overestimate the PSD. Some studies have reported a reasonable correlation between the CAK value and the PSD.^38^, ^39^ DAP is also a general predictor of the PSD in percutaneous IR procedures. There are many studies on the correlation coefficient between the DAP and the PSD.^38^, ^40^, ^41^ In a clinical site, the PSD can be estimated from the CAK value or DAP. Moreover, dosimeters are available for real‐time monitoring of surface doses for individual IVR patients.^12^, ^22^, ^23^, ^33^, ^34^, ^35^, ^42^ Thus, the estimation of surface doses, such as the PSD or ESD, can be readily performed. However, it is difficult to estimate organ‐specific doses including the fetal dose inside the body using the CAK value, DAP, or actual measurements. In this study, the fetal dose obtained using the dose calculation software was confirmed to be approximately equal to those obtained via actual measurements. The results of previous studies^20^, ^26^ suggest that when an MIRD phantom is used with conventional dose calculation software, the values obtained differ from those obtained when an anthropomorphic phantom is used to represent the anatomy of a woman in late pregnancy. Given that the VirtualDose‐IR software used in this study utilized a set of anatomically realistic pregnant patient phantoms, its accuracy is expected to be high compared with that of conventional dose calculation software. Although differences in the radiation dose may occur because of fetal size and location, the highest organ doses inside the body were received by the gonads (14.87 mGy and 14.73 mGy) in this study, and the fetal doses were 2.39 mGy and 1.89 mGy. A conservative estimate of the lifetime risk of radiogenic induction of childhood cancer or leukemia is approximately one in 170 for 100 mGy.^3^ For fetal doses less than 100 mGy, there is no medical justification for terminating a pregnancy because of radiation exposure.^43^ Therefore, the placement of IVC‐Fs in pregnant patients has more benefits than risks and should be performed when clinically indicated.

The previous study^32^ compared patient doses reported by VirtualDose‐IR with Monte Carlo simulations for various beam angles. VirtualDose‐IR results were in good agreement with Monte Carlo results for most organs under all the beam angles. This study only evaluated a posterior‐anterior beam entry. From the previous study^32^, we expect to maintain accuracy between our actual and software results with varying beam entry angles. However, the fetal dose difference may be the maximum between actual and software results because swinging beam entry angles may result in a portion of the fetus being outside the irradiation field, as already discussed. The user should ensure that the range of fetus is within the irradiation field.

This study inherently bears some limitations. The fetal lie, presentation, and position vary amongst patients. The software assumes the fetus is longitude lie‐cephalic presentation‐occiput anterior and cannot change the fetal lie, presentation, and position. The quoting of the fetal organ doses on the different fetal lie, presentation, and position from the software may introduce individual uncertainties for radiation dose calculations for these organs. Furthermore, different voltages were used in actual measurements and software calculations because of the limited tube voltage of choices in the dose calculation software. With increasing differences in tube voltage settings, the results of radiation doses are affected. On an X‐ray examination, Kawasaki et al.^44^ reported the ESD and organ dose varied by a factor of approximately 0.8 with the change in the tube voltage from 100 to 90 kV at a constant milliampere‐second value and focus‐to‐film distance. As the differences in tube voltages used in this study were 4 and 7 kV, the results could vary by a factor of 0.8 or less.

Because the radio‐sensitivity and radiation risks of developing fetal organs vary across different gestational ages, organ‐scale radiation dosimetry for the fetus is crucial and highly desired for epidemiological studies aiming to correlate between conceptus radiation exposure and organ‐specific childhood cancer after birth. Although there were some limitations to this study, the fetal organ dose across different gestational ages is useful.

## 5 CONCLUSION

A dose calculation software can be used to evaluate the fetal radiation dose and ESD in pregnant women. However, in the case of the former, the user should be careful to ensure how much of the fetal length is inside the irradiation field to prevent underestimation or overestimation.

## AUTHOR CONTRIBUTIONS

Y. Matsunaga PhD, contributed to the design and implementation of the research,to the analysis of the results, and to the writing of the manuscript. T. Haba PhD, was involved in methodology of radiation dose measurements in phantom study. M. Kobayashi PhD, was involved in methodology of radiation dose measurements. S. Suzuki PhD, was involved in methodology of radiation dose measurements and the analysis of the results. Y. Asada PhD, was involved in methodology of radiation dose measurements. K. Chida PhD, was involved in methodology, review, and editing.

## CONFLICT OF INTEREST

The authors have no conflict of interest to disclose. The submitted manuscript does not contain previously published material and is not under consideration for publication elsewhere.

## Data Availability

The datasets generated and/or analyzed during the current study are available from the corresponding author on reasonable request.
